# Knowledge and Attitude Toward Human Papillomavirus (HPV) and Its Vaccination in the Saudi Population: A Systematic Review

**DOI:** 10.7759/cureus.103427

**Published:** 2026-02-11

**Authors:** Eman Alshehri, Khalid M Akkour, Ahmed Sherif Abdel Wahab, Ghadeer K Al-Shaikh, Nada Alayed, Salwa Neyazi, Ahmad Almalki, Mustafa Smisim, Mohammed Abdelrazeq, Marwan Gamal

**Affiliations:** 1 Obstetrics and Gynecology Department, Faculty of Medicine, King Saud University, Riyadh, SAU; 2 Obstetrics and Gynecology Department, King Saud University Medical City, Riyadh, SAU; 3 Obstetrics and Gynecology Department, Faculty of Medicine, Ain Shams University, Cairo, EGY; 4 Obstetrics and Gynaecology Department, Faculty of Medicine, King Saud University, Riyadh, SAU; 5 College of Medicine, King Saud University, Riyadh, SAU

**Keywords:** cervical cancer, human papilloma virus, review, saudia arabia, vaccine

## Abstract

Human papillomavirus (HPV) vaccination represents an important public health opportunity in Saudi Arabia. This systematic review aimed to evaluate knowledge, attitudes, and uptake regarding the HPV vaccine among various demographic groups in the country. Following the Preferred Reporting Items for Systematic Reviews and Meta-Analyses (PRISMA) guidelines, a comprehensive search of databases, including PubMed/MEDLINE, Embase, Web of Science, Scopus, Cochrane Library, and PsycINFO, was conducted from inception through December 2025. The review included studies providing quantitative data on HPV vaccine knowledge, attitudes, beliefs, or uptake. A total of 28 studies published between 2014 and 2024 were analyzed, including studies involving parents, students, and healthcare professionals. Findings revealed a notable prevention paradox: while reported willingness to vaccinate was moderate to high (29.1-87.9%), actual vaccination uptake was consistently low (1-23%). Significant gaps in knowledge were identified, with limited awareness of HPV's association with cervical cancer and optimal vaccination timing. Factors such as higher education and healthcare employment predicted greater acceptance, whereas barriers included safety concerns, insufficient provider engagement, and sociocultural influences. The review concludes that suboptimal HPV vaccine uptake stems largely from systemic and informational gaps. A coordinated national strategy, featuring public education, healthcare provider training, and programmatic interventions such as school-based vaccination, is urgently needed to improve coverage and reduce HPV-related cancer burden.

## Introduction and background

Human papillomavirus (HPV) infection represents a significant global public health burden, constituting the most common sexually transmitted infection and a necessary cause of several cancers, including cervical, oropharyngeal, and anogenital malignancies [1]. While effective prophylactic vaccines have been available for nearly two decades, their uptake remains suboptimal in many regions, including the Middle East. In Saudi Arabia, despite the introduction of the HPV vaccine, vaccination coverage has consistently fallen short of public health targets, indicating a critical gap between vaccine availability and population acceptance.

Previous research has begun to explore this challenge. A recent systematic review by Alshahrani et al. (2024) [2] analyzed perspectives on HPV vaccination across Gulf Cooperation Council (GCC) countries, identifying overarching themes of low awareness and safety concerns among parents. Furthermore, a comprehensive meta-analysis by Gebreal et al. (2025) [3] quantified a significant "intent-action gap" throughout the Eastern Mediterranean Region, revealing a stark disparity between high parental willingness (61%) and critically low actual vaccination uptake (7%). While these reviews provide valuable regional context, they leave several key questions unanswered, specific to the Saudi population. The GCC review [2] focused solely on parents, while the EMR analysis [3], although including Saudi studies, aggregated data across 12 nations with diverse healthcare systems and cultural landscapes, potentially obscuring nation-specific determinants.

Therefore, a comprehensive, Saudi-specific synthesis is urgently needed to inform targeted national policy. This systematic review aims to address this gap by providing a nuanced analysis of the knowledge, attitudes, and uptake of the HPV vaccine across the diverse demographics of Saudi Arabia. Guided by the PICO framework, this review seeks to answer the question: "What is the level of understanding and the general attitudes towards Human Papillomavirus (HPV) and its vaccination within the general population and specific subgroups in Saudi Arabia?"

Unlike previous regional efforts, this review will (1) analyze data from a wider array of demographic groups (including students, healthcare professionals, and patients, alongside parents); (2) quantify the precise "know-do gap" within the Kingdom; and (3) identify the specific hierarchy of barriers and predictors unique to the Saudi context. By doing so, this review provides the granular evidence necessary to develop targeted, multi-level interventions to improve HPV vaccination rates and ultimately reduce the burden of HPV-related cancers in Saudi Arabia.

## Review

Materials and methods

Review Question and PICO Framework

This review addressed the question: "What is the level of understanding and the general attitudes towards human papillomavirus (HPV) and its vaccination within the general population and specific subgroups in Saudi Arabia?" The PICO framework was developed to guide the process (E.A., K.A., and A.S.A.H.).

Search Strategy

A comprehensive search was conducted from inception to December 31, 2025, across PubMed/MEDLINE, Embase, Web of Science, Scopus, Cochrane Library, and PsycINFO (M.A. and M.M.G.). The strategy utilized a mix of MeSH terms and keywords related to HPV, vaccines, knowledge/attitude, and Saudi Arabia, combined with Boolean operators (M.A. and M.M.G.). Reference lists of all included studies and pertinent review articles were manually searched to identify additional eligible research (M.N.S. and A.A.).

Study Selection (Screening) Process

The study selection process consisted of two phases, carried out by two independent reviewers (N.A. and S.N.). In Phase 1, titles and abstracts of retrieved citations were screened against established eligibility criteria. Phase 2 involved a thorough assessment of the full texts of all studies deemed potentially relevant for final inclusion. Any disagreements between the reviewers were resolved through discussion or by involving a third reviewer (G.A.S.), and this was documented using a PRISMA flow diagram [4].

Eligibility Criteria

The eligibility criteria were defined as follows: observational studies, including cross-sectional, cohort, and case-control designs, must be conducted in Saudi Arabia and should provide data concerning knowledge or attitudes related to HPV and its vaccine. Studies that do not take place in Saudi Arabia, those focusing solely on clinical outcomes, as well as reviews, commentaries, editorials, and studies that are exclusively qualitative, were excluded from consideration.

Data Extraction

Data were independently gathered by two reviewers (M.N.S. and A.A.) utilizing a standardized form. The information extracted encompassed various aspects, including study characteristics like author, year, region, and design; population details such as sample size and demographics (for instance, whether participants were parents or students); methodology, including the data collection instrument used; and key outcomes reflecting knowledge levels and attitudes, represented through percentages and themes.

Quality Assessment (Risk of Bias)

The methodological quality of included studies was assessed independently by two reviewers (N.A. and S.N.) using the Joanna Briggs Institute (JBI) Critical Appraisal Checklist for Analytical Cross-Sectional Studies. Disagreements were resolved by consensus or by a third reviewer (G.A.S.).

Data Synthesis

Due to the anticipated heterogeneity in study populations, measurement tools, and outcome reporting, a narrative synthesis was conducted (A.S.A.H., G.A.S., E.A., and K.A.). The findings were structured around the main outcomes (knowledge and attitudes). Results were summarized descriptively and presented in structured tables to compare and contrast findings across different population subgroups and study characteristics. This thematic summary identified key patterns, major barriers and facilitators, and gaps in the existing evidence base within the Saudi context.

Results

**Figure 1:**
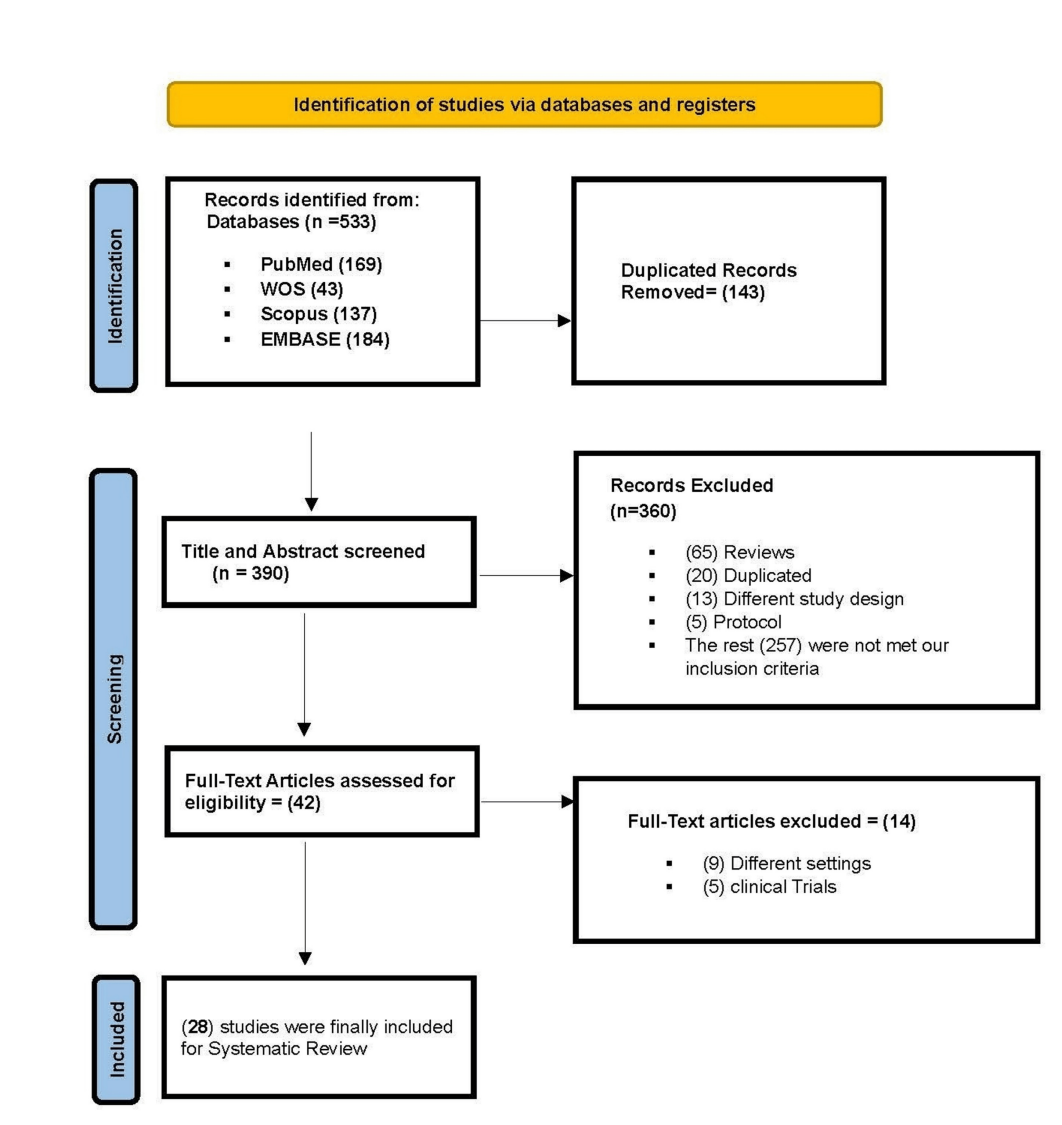
Preferred Reporting Items for Systematic Reviews and Meta-Analyses (PRISMA) flow diagram of study selection Reference: [4]

The systematic search and selection process, detailed in the PRISMA flow diagram (Figure 1) [4], identified 28 studies meeting the inclusion criteria for this review [3-30].

Study Selection

The initial database search yielded 533 records. After the removal of 143 duplicates, 390 titles and abstracts were screened. Following a detailed eligibility assessment of 42 full-text articles, 28 studies were included for final synthesis. The complete selection process is illustrated in Figure 1.

Characteristics of the Included Studies

The characteristics of all 28 included studies are summarized in Table 1. All studies were published in English between 2014 and 2024 and conducted within Saudi Arabia. Geographically, research was focused on major urban centers, with the highest number of studies conducted in Riyadh (n = 7) [3,4,9,10,21,23,28], followed by nationwide studies (n = 5) [7,12,13,24,25] and Makkah (n = 4) [11,16,27,28].

**Table 1:** Characteristics of the included studies on HPV knowledge, attitudes, and vaccination in Saudi Arabia HPV: human papillomavirus, CC: cervical cancer, KSA: Kingdom of Saudi Arabia, HBM: Health Belief Model, MOH: Ministry of Health

Study (year)	Region	Population	Sample size (N)	Design	Data collection	Key knowledge findings	Vaccine acceptance (%)	Actual uptake (%)	Primary barriers	Key predictors of acceptance
Al-Shaikh et al. (2014) [4]	Riyadh	Female university students (health colleges)	1258	Cross-sectional	Self-administered	• 95.7% had poor knowledge. • Only 10.9% knew vaccine contains HPV. • 26.5% knew that HPV is a risk factor. • 59.6% knew that STDs are a risk factor.	Not explicitly reported	Not explicitly reported	• Worry about side effects (51.9%) • Fear of injection (26.5%) • Time constraints (20.4%) • Cost (13.3%) • Family refusal (8.4%) • Belief that cancer is too rare (9.2%)	• Higher year of study (p = 0.002) • College (Medicine/Pharmacy had higher scores, p = 0.001).
Al-Shaikh et al. (2014) - Educational Intervention [5]	Riyadh	Female healthcare students	535	Pre-post intervention	Self-administered (Pre/Post-test)	Significant knowledge improvement (p < 0.01 for most items): • Knowledge that vaccine contains HPV: 13.6% → 37.2% • Knowledge that HPV is a risk factor: 28.4% → 54.8% • Knowledge that cervical cancer is preventable: 50.3% → 76.6%	Not directly reported	Not reported	Barriers significantly reduced (p < 0.01): • Worry about side effects: 52.5% → 29.2% • Fear of injection: 24.3% → 13.8% • Cost concerns: 13.6% → 6.7%	Highly effective educational intervention: • Significant knowledge increase across all domains • Significant reduction in perceived barriers
Malibari (2018) [6]	Not specified	Women (general public)	412	Cross-sectional	Self-administered	• HPV vaccine awareness: 10.5% • HPV awareness: 16.4% (of those who knew CC) • 78.6% knew about cervical cancer • 75.6% cited social media as info source.	Willingness (if aware): 96.3% (n = 27)	Not reported	For screening (proxy for barriers): • Lack of knowledge (17.9%) • Embarrassment/shyness (14.3%) • Waiting for doctor's recommendation (66.7%)	• Higher education trend (p = 0.274) • Significant association with marital status (p = 0.023) and type of work (p = 0.047)
Anfinan (2019) [7]	Not specified (nationwide implied)	Physicians	404	Cross-sectional	Online/self-administered questionnaire	• Knowledge score: 65% (average) • 43.1% knew the recommended age for vaccination. • 57.4% knew that the vaccine is effective for boys and girls. • 34.2% were aware of the national immunization program inclusion.	Not directly applicable (study focused on physician knowledge and opinions, not personal acceptance)	Not applicable	Barriers to recommendation: • Lack of knowledge (37.1%) • Concern about side effects (22.8%) • Not part of national program at the time (18.8%) • Cultural/religious concerns (8.9%)	Strong predictors of knowledge: • Specialty (gynecologists had the highest knowledge.) • Younger age (<40 years) • Working in the private sector
Anfinan (2019) [8]	Nationwide (multiple regions)	Physicians	2000	Cross-sectional	Online/self-administered questionnaire	• Adequate knowledge (score ≥8): 62% • Ob/Gyns had the highest knowledge (78.2%). • Non-Ob/Gyns had significantly lower knowledge (39.1% adequate). • Residents had the lowest knowledge level (48% adequate).	• Personal acceptance: 41.2% • Parental acceptance (for children): 77.6% • Support for national program: 69.6%	7.6% (personal uptake)	Reasons for refusal (top 3): 1. Not at risk for HPV (58.5%) 2. Lack of knowledge (21.1%) 3. Not sexually active (14.7%) • Side effects concerns (8.4%) • Cost/reimbursement (8.7%)	Strong predictors of positive attitude: • Younger age (<40 years) • Adequate knowledge • Ob/Gyn specialty • Working in Central region • Being a resident (junior practice level)
Almazrou et al. (2020) [9]	Not specified	Physicians (pediatricians and family medicine)	173	Cross-sectional	Self-administered questionnaire	• Good cervical cancer knowledge: 61.3% • Good HPV knowledge: 58.4% • 84.4% knew that HPV vaccine exists. • 47.4% mistakenly believed that Pap smear is not needed post-vaccination or were unsure. • 44.5% knew HPV 6/11 association. • 57.2% knew HPV 16/18 association.	• Personal acceptance: Not directly reported • Parental acceptance (for daughter): 82.1% • Recommendation for patients: 66.5-87.9% (varies by age)	3.5% (personal uptake)	Attitudinal/practice barriers: • 42.8% never discuss sexual health with patients. • 20.2% believe vaccine encourages early sexual activity. • 13.3% lack confidence in vaccine safety. • 18.5% believe patients not at risk for HPV.	Predictors of better knowledge: • Family Medicine specialty (vs. Pediatrics) • ≥10 years of practice • Lack of parental awareness perceived as a significant barrier (OR = 2.01)
Alrajeh and Alshammari (2020) [10]	Riyadh	Female patients (primary care clinics)	326	Cross-sectional	Self-administered questionnaire	• Extremely low knowledge: 41.1% scored zero (no correct answers). • Mean knowledge score: 2.5/14 (±3.0) • HPV causes cervical cancer: 20.6% • HPV vaccine exists: 21.5% • Vaccine protects against cervical cancer: 24.2% • Vaccine age awareness (9+ years): 13.5%	Parental acceptance (for daughters): 29.1%	3.4% (personal uptake)	• Severe lack of awareness (primary barrier) • 57.1% did not know where to get vaccine info. • 57.7% did not know where to get vaccinated.	Strong predictors of better knowledge: • Positive attitude toward daughter vaccination (p < 0.0001) • Undergoing Pap smears (p = 0.002) • (Demographic factors not significant)
Azer et al. (2020) [11]	Riyadh	University students (medical vs. non-medical)	172 (86 + 86)	Cross-sectional comparative	Self-administered questionnaire	Massive knowledge gap: • 96.5% medical students knew that a vaccine exists vs. 20.9% of non-medical students. • 96.5% medical students knew that CC is preventable vs. 36% of non-medical students. • 83.7% medical students knew that a vaccine is available in KSA vs. 14% of non-medical students. • Both groups had poor knowledge on specifics (efficacy, condom use post-vaccine).	Personal acceptance: • Medical: 60.5% • Non-medical: 26.7%	Not reported	Top barriers: • Non-medical: Inadequate information (67.4%) • Medical: Vaccine availability (47.7%) • Cost concerns (7-8%) • Worry about efficacy (1.2-11.6%)	Strongest predictor: • Being a medical student (p < 0.001 for most knowledge/acceptance items) • Gender differences were generally not significant.
Gari et al. (2022) [12]	Makkah	Female university students (medical vs. non-medical)	479	Cross-sectional	Online structured questionnaire	• 85% heard of cervical cancer. • 59.3% heard of HPV. • 69.5% heard of HPV vaccine. • 44.9% heard of Pap smear. • Only 16.5% knew the optimal vaccine age ( nine to 13 years). • Only 6% knew the vaccination frequency (three doses/six months). • 59.7% knew that cervical cancer is preventable.	Personal acceptance: 72%	2.1% (personal uptake)	• Did not hear about vaccine (17.3%) • Worry about side effects (11.5%) • Not believing in benefits (8.4%) • Vaccine unaffordable (4.2%)	• Age 22-25 years (p < 0.05) • Medical specialty (p < 0.05) • Previous knowledge of cervical cancer/HPV • Previous Pap smear • Personal/relative history of cervical cancer
Ibrahim et al. (2022) [13]	Najran	Women (general population)	1085	Cross-sectional	Questionnaire-based (Health Belief Model)	• Health Belief Model findings: 41% had low perceived susceptibility to CC. 70.4% had high perceived seriousness of CC. 28.4% had high barriers to Pap smear. 85.3% had high perceived benefits of Pap smear. 79% had high health motivation.	Not explicitly reported	1.0% (HPV vaccine uptake); 2.0% (Pap smear screening)	• Barriers to Pap smear (28.4% reported high barriers) • Low perceived susceptibility (41% low) • Lack of screening history (98% never had Pap smear)	• Significant correlations between all HBM constructs (p < 0.05) • Demographic factors: married (91.7%), urban (93.9%), university education (65.3%) • 47.6% history of genital infection • 74.6% history of contraceptive use
Barhamain and Alwafi (2022) [14]	Nationwide (multiple regions)	Women (general population)	609	Cross-sectional	Online questionnaire	• 54.8% have insufficient knowledge about HPV. • 37.9% have insufficient knowledge about HPV vaccine. • 53.6% learned about HPV through the Internet/social media. • 55.8% did not know if they are prone to HPV infections. • 74.9% considered health practitioners as a good knowledge source.	Personal intention: Not explicitly reported. Parental acceptance (for daughters): 86.5%	3.0% (personal uptake)	• Lack of vaccine awareness (64.0%) • Vaccine not available in PHC (16.6%) • Fear of side effects (3.4%) • Low perceived risk (3.4%) • High price (0.7%)	• Medical education/career (p = 0.049 for uptake, p = 0.007 for daughter vaccination) • Better knowledge levels (p < 0.001 for both domains) • Higher education (85.1% bachelor+) • Urban residence (93.9%) • Married (63.5%)
Alhusayn et al. (2022) [15]	Not specified	Parents	296	Cross-sectional	Questionnaire-based	• High education level (84.8% higher education) • 100% Saudi nationals • 82% female respondents (mothers) • Strong trust in MOH information (79.6%) • Awareness significantly associated with vaccine uptake	Planning to vaccinate: 44% Future vaccination consideration: 26.6%	10.5% (received vaccine for self/children) • 36.7% received 1 dose • 30% received 2 doses • 33.3% received 3 doses	• Did not hear about vaccine before (68.1%) - primary barrier • Lack of awareness • Hesitancy among unvaccinated	• Employment status (p = 0.005) • Age 31-40 years (p < 0.001) • Higher education level (p = 0.02) • Vaccine awareness (p < 0.001) • Trust in MOH information (p < 0.001) • Doctor recommendation (26.8% cited) • Belief in vaccine safety (65.8% cited)
Alkalash et al. (2022) [16]	Western Region	Parents	343	Cross-sectional	Questionnaire-based	• 65.3% had no knowledge about HPV. • 67.1% had no knowledge about HPV vaccine. • 94.2% had a poor knowledge level. • 32.7% knew that HPV causes cervical cancer. • 59.5% did not know the harm associated with HPV. • 38% learned about vaccine from physicians. • 29.2% from the Internet • 25.8% from social media	Willing to vaccinate children: 58.6%. Maybe: 26.2%. Refusal: 15.2%	7.2% (children vaccinated) • Among those with female children	• Not at risk of infection (75.2%) • Lack of knowledge about vaccine (13.4%) • Do not believe in effectiveness (7.6%) • Fear of side effects (1.9%)	• Married status (p = 0.011) • University education (p = 0.043) • Knowledge from physicians (p = 0.001) • Employment status (p = 0.028) • Conviction about vaccine effectiveness (p = 0.004) • Access to vaccination book (p = 0.011)
Turki and Alqurashi (2023) [17]	Makkah	Adult women (primary care centers)	534	Cross-sectional	Questionnaire-based	• Knowledge levels: poor (28.8%), moderate (33.3%), and high (37.8%) • 6.0% had been tested/screened for HPV. • 46.4% had questions about vaccine availability/safety/efficiency. • 82.8% felt the need for educational sessions.	Personal acceptance (free): 65.5%. Personal acceptance (cost): 37.5%. Parental acceptance (free): 60.3%. Parental acceptance (cost): 42.7%	Not explicitly reported	• Fear of injections (27.7%) • Cost concerns (23.2%) • Family/community refusal (9.7%) • Lack of risk perception (80.1% did not feel at risk)	• Higher education (p < 0.001) • Higher income (p < 0.001) • Employment status (p < 0.001) • Age 21-40 years (p = 0.002) • Saudi nationality (p = 0.014) • Married status (p = 0.029) • Recommendation willingness (54.3%)
Alnaeem et al. (2023) [18]	Eastern Region	Parents	380	Cross-sectional	Questionnaire-based	• Mean knowledge score: 3.00/11 (SD = 2.9) • 62.9% doctors did not mention HPV vaccine. • 28.4% had lower/no knowledge. • 71.6% had higher knowledge. • Significant knowledge gaps in general HPV information.	Parental acceptance (for children by age 12): 41.1%. Spousal acceptance: 25.6%	7.2% (children vaccinated)	• Lack of doctor recommendation (62.9%) • Spousal disagreement (34.7% disagreed, 39.7% unsure) • Age (>50 years had lower acceptance) • Lower education levels	• Female gender (p<0.001) • Higher education (p=0.045) • Employment status (p<0.001) • Age 18-50 years (p<0.001) • Having 3-4 children (p=0.015) • HPV awareness (p<0.001) • MOH support (38.5% cited) • Belief in vaccine effectiveness (35.1% cited) • Physician advice (8.1% cited)
Tobaiqy et al. (2023) [19]	Al-Madinah region (primarily)	General population (parents/adults)	721	Cross-sectional	Online questionnaire	• 59.4% heard about HPV. • 37.4% knew that HPV causes cervical cancer. • 37% knew that HPV is sexually transmitted. • 54.4% heard about the HPV vaccine. • 32.7% knew that the vaccine prevents cervical cancer. • 47.5% knew that the vaccine is available in the country. • 76.5% did not know about side effects.	Personal willingness: 63.9%. Recommend for child (nine to 12 years): 36.3%. Recommend for friends/relatives: 67.7%	3.1% (personal uptake)	• Lack of awareness (57.3%) • Concern about vaccine safety (29.4%) • Fear of needles/injections (17.5%) • No time (15.3%) • Family refusal (7.1%) • Cost (4.2%) • Cultural/religious beliefs (5.4%)	• Higher education level (p = 0.002) • Younger age (18-25 years: 49.2% good knowledge) • Information sources: social media (35.1%), Internet (30.8%), doctors (26.6%) • 50.2% wanted more information before vaccination • 47.9% supported school education on HPV • 55.2% supported national immunization program inclusion
Alshehri et al. (2023) [20]	Not specified	Parents	773	Cross-sectional	Questionnaire-based	• 35.4% heard about HPV. • 31.8% heard about HPV vaccine. • 46.1% had poor knowledge. • 38.5% had good knowledge. • 26% knew that HPV causes cervical cancer. • 17.6% had been informed by the medical team. • 69.1% unsure about HPV-cancer link.	Intention to vaccinate daughters: 44.5%. Not sure: 43.6%. No intention: 11.9%. Willing if freely available: 34.8%	Not explicitly reported (child vaccination)	• Lack of awareness (primary barrier) • Uncertainty about benefits (54.1% unsure) • Concerns about long-term effects (69.4% unsure) • Vaccine perceived as "too new" (52.1% wanted to wait) • Limited medical information (only 8.5% informed by medical team)	• Employment status (p = 0.032) • Higher knowledge level (p = 0.000 correlation) • Fathers vs. mothers (p = 0.002) • Non-Saudi nationality (p = 0.007) • Higher education (p = 0.001) • Higher income (p = 0.014) • Healthcare sector employment (p = 0.014)• Internet as main info source (46.4%) • Health teams as info source (25.7%)
Maqbul et al. (2024) [21]	Not specified	Parents	424	Cross-sectional	Questionnaire-based	• 47.2% knew that HPV is sexually transmitted. • 53.8% knew that HPV causes cervical cancer. • 32.1% knew that Pap smear screens for cervical cancer. • 43.2% knew that HPV causes genital warts. • 39.6% knew that HPV is often asymptomatic. • 40.8% knew that smoking increases cervical cancer risk. • 56.8% are unsure if antibiotics treat HPV. • Significant knowledge gaps exists by gender (all p < 0.05).	Strongly believe in HPV vaccine value: 51.7%. Agree that vaccine prevents cervical cancer: 47.2%. Agree that vaccine prevents genital warts: 47.2%.	Not explicitly reported	• Concerns about side effects (67.0%) • Uncertainty about vaccine benefits (48.3% disagreed/unsure about wart prevention) • Lack of knowledge about HPV seriousness (30.9% disagreed/unsure) • Gender differences in perceptions (mothers vs. fathers)	• Female gender (mothers had better knowledge, p < 0.05) • Higher education (73.3% university) • Belief in cervical cancer seriousness (70.5%) • Recognition of HPV as serious health problem (64.6%) • Previous vaccination compliance (67.5% gave all childhood vaccines)
Bin Alamir et al. (2024) [22]	Not specified	Parents	281	Cross-sectional	Questionnaire-based	• 51.1% males, 57.3% females did not know that HPV infections are rare. • 34% males, 59.8% females did not know that men can get HPV. • 42.6% males and 59.4% females knew that HPV can be asymptomatic for years. • 48.9% males and 59.8% females did not know that vaccine protects against genital warts. • 51.1% males and 72.6% females knew that vaccinated can still get cervical cancer. • Significant gender differences in knowledge (p < 0.05 for several items).	Support compulsory vaccines: 71.8% females, 72.3% males Belief in vaccine choice: 71.4% females, 57.4% males (p = 0.046)	Vaccine refusal for daughters: 25.6% females, 21.3% males	• COVID-19 vaccine controversy reduced confidence (41% females, 44.7% males) • Concerns about past complications (35.5% females, 48.9% males) • Difficulty discussing vaccine with daughters (23.1% females, 55.3% males) • Preference for natural immunity (40.2% females, 51.1% males).	• Married status (p = 0.017 for vaccine refusal) • Gender differences in attitudes and knowledge • Knowing someone ill from not vaccinated (p = 0.013) • Previous Pap smear experience (p = 0.048) • Information seeking behavior (44.4% females searched info)
Alherz et al. (2024) [23]	Riyadh	Parents of daughters	390	Cross-sectional	Self-administered questionnaire	• Cervical cancer awareness: 78.7% • HPV awareness: 50.0% • HPV vaccine awareness: 41.3% • The Internet was the primary source of information (41-42% across all topics).	Parental acceptance (for daughter): 50.8%	Not reported	• Severe lack of awareness (primary barrier) • Low baseline knowledge of HPV and the vaccine	• Sex: Female parents had significantly higher awareness of cervical cancer (p = 0.000). • Education: Higher education level significantly predicted awareness of HPV (p = 0.043) and the HPV vaccine (p = 0.001). • Age and marital status were not significant predictors of awareness.
Aldawood et al. (2024) [24]	Riyadh	Male and female college students (medical fields)	271	Pre-post intervention	Self-administered questionnaire	• Pre-intervention: Poor baseline knowledge (mean scores: 3.13/18 for males, 5.55/18 for females) • Post-intervention: Significant knowledge improvement across all demographics (p < 0.0001 for all) • Overall knowledge gain: +9.0 points (Mean: 4.8 → 13.8) • Pre-intervention awareness of HPV: 57.6% (35.2% male, 65.5% female)	Not reported	Not reported	• Primary barrier: Extremely low baseline knowledge • Significant knowledge gap between genders (Female > Male, p<0.05) • Lower GPA and younger age associated with lower pre-intervention knowledge	• Effective educational intervention: highly effective at improving knowledge (p < 0.0001) • Gender: Female gender was a predictor of higher pre-intervention awareness (p < 0.05). • All demographic groups showed significant knowledge gain, with the largest improvements in those with the lowest baseline scores (e.g., males, lower GPA students.
Bakhashab et al. (2024) [25]	Nationwide (Saudi Arabia)	Parents/guardians of children	386	Cross-sectional	Online/self-administered questionnaire	• HPV awareness: 64.0% • HPV vaccine awareness: 33.7% • Knowledge gaps: 53.4% unsure how HPV spreads; 49.5% had "not enough information" on HPV-related diseases. • Misconceptions: 37.0% believed vaccine not in schedule; only 6.5% knew it's for both genders. • Primary info source: Internet/social media (48.2%)	Parental acceptance (for child): 56.0% (strongly agree/agree to vaccinate)	Child uptake: 8.5% (11.7% unsure)	• Lack of information (primary barrier; 15.3%) • Fear of side effects (13.7%) • Low perceived risk (child not at risk: 4.7%) • Lack of physician recommendation (only 19.9% received one)	• High general vaccine confidence (88.1% agree vaccines are effective) • Key information needs: vaccine safety (20.2%), general HPV info (17.4%), efficacy (16.6%) • Trusted sources for info: healthcare providers (pediatricians 34.5%, family physicians 40.9%)
Algaadi et al. (2024) [26]	Nationwide (multiple regions)	General public (primarily students)	516	Cross-sectional	Online/self-administered questionnaire	• Overall: 43.7% had good knowledge, 56.3% had poor knowledge. • Awareness: 41.5% knew that HPV causes cervical cancer. • Transmission: Only 35.9% knew that HPV is sexually transmitted. • Susceptibility: 39.9% correctly identified that both genders can be infected. • Asymptomatic nature: 94.0% knew that someone can be infected and not know.	Not explicitly reported (% agree/strongly agree with vaccine effectiveness: 82.4%)	Not reported	• Primary barrier: Widespread knowledge gaps and misconceptions • Concern about side effects: 70.0% (agree/strongly agree) • Low perceived relevance (only 30.2% knew that HPV is common)	• Gender: Female gender was a significant predictor of better knowledge (p = 0.023). • Education: Higher education level significantly predicted better knowledge (p = 0.003). • Occupation: Healthcare professionals and students had significantly better knowledge (p = 0.001). • Nationality, age, and region were not significant predictors.
Alshrari (2024) [27]	Northern Border Province	General public	1041	Cross-sectional	Self-administered questionnaire	• Extremely low awareness: Only 37.9% had heard of HPV. • Disease linkage: 25.4% knew that HPV causes cervical cancer. • Transmission: Only 22.0% had any idea how HPV is transmitted. • Complications: Very low awareness of genital cancer (8.6%) and warts (8.1%). • Prevention: 25.4% identified vaccination as a preventive measure.	Not explicitly quantifiable (%); study focused on barriers rather than acceptance rates	Not reported	• Severe lack of information/awareness (primary barrier) • Concerns about safety/complications • Misconceptions: 13.8% erroneously believed that antibiotics prevent HPV. • Cost and vaccine availability concerns • Perception of being "too old" for vaccination	• Gender: Female gender was a strong, significant predictor of better knowledge across multiple domains (p < 0.001). • Educational status: Higher educational status was a significant predictor of HPV awareness (p < 0.05). • Strong support for vaccinating both genders (among those aware).
Tobaiqi et al. (2024) [28]	Medina Region	Women	721	Cross-sectional	Self-administered questionnaire	• Overall: Only 24.3% had good knowledge/awareness. • HPV awareness: 59.4% had heard of HPV. • Disease linkage: 37.4% knew that HPV causes cervical cancer. • Transmission: 37.0% knew that HPV is sexually transmitted. • Vaccine awareness: 54.4% had heard of HPV vaccine. • Primary info source: Social media (35.1%) > Healthcare professionals (26.6%)	Personal acceptance: 63.9% (willing to receive). Recommend for others: 67.7%. Recommend for children: 36.3%	3.1%	• Lack of awareness (primary barrier; 57.3%) • Concern about vaccine safety (29.4%) • Fear of needles/injections (17.5%) • No time (15.3%) • Family refusal (7.1%) • Need for more information (70.2% agree/strongly agree)	• Education: Higher education level (Master's/Doctoral) significantly predicted better knowledge (p = 0.002). • Income: Higher monthly family income (>20,000 RS/month) significantly predicted better knowledge (p < 0.0001). • Age, marital status, occupation, and region were not significant predictors.
Azzi et al. (2024) [29]	Nationwide (primarily central region)	Women	858 (combined 2022 and 2024 datasets)	Cross-sectional (comparative)	Online/self-administered questionnaire	• Overall HPV awareness: 49.4% • HPV vaccine awareness: 38.0% • Significant improvement: Awareness increased from 2022 (38.8%) to 2024 (71.7%) • Primary info source: Social media (63.2%) • Pap smear uptake: 25.8% had undergone screening. • Vaccine knowledge gaps: Only 8.7% are aware of vaccine for males, 12.5% for children.	Personal acceptance (if free): 67.6% child acceptance (if free): 36.0%	Personal uptake: 1.5% (full/partial). Child uptake: 2.2%	• Lack of information (primary barrier; 57.9%) • Fear of side effects (21.9%) • Perceived unnecessary (18.3%) • Low awareness of free MOH program (67.8% unaware) • Access: 80.1% did not know where to get vaccinated	• Higher education: Bachelor's degree or higher significantly predicted awareness (p = 0.049 in 2022, p = 0.038 in 2024). • Higher income: >10,000 SAR monthly income significantly predicted awareness in 2024 (p < 0.001) • Central Region residence: Significant predictor of awareness in 2024 (p = 0.026) • Awareness itself strongly predicted willingness to vaccinate (p = 0.001-0.005).
Alshahrani et al. (2024) [30]	Asir	Parents of minors	539	Cross-sectional	Self-administered questionnaire	• Major knowledge gaps: Only 34.6% knew that HPV causes cervical cancer; 47.9% knew that vaccine prevents cervical cancer. • Vaccine specifics: Only 34.6% knew recommended age (9-15 years); 21.7% knew it should be given before the first intercourse. • Safety: Only 36.3% believed that vaccine is safe. • Program awareness: 66.8% knew that vaccine is offered free by the MOH.	Parental acceptance (for daughter): 65.9%	Not explicitly reported	• Poor knowledge about HPV, cervical cancer, and vaccine details • Safety concerns • Lack of awareness about vaccine schedule and recommendations • Misconceptions about vaccine causing infection (7.6-10.3%)	• Higher knowledge: Highest knowledge tertile significantly predicted acceptance (OR = 2.78, p = 0.002) • Healthcare worker status: Associated with acceptance in the bivariate analysis (p = 0.002) • Social influence: Friend/relative vaccination strongly motivated acceptance (OR = 6.75, p < 0.001) • Official recommendation: MOH recommendation strongly motivated acceptance (OR = 6.07, p < 0.001) • Number of daughters: Having one daughter (vs > 2) predicted acceptance (OR = 2.25, p = 0.016)
Bakhsh et al. (2024) [31]	Multi-center	Female medical students	246	Cross-sectional	Self-administered questionnaire	• General HPV awareness: 82.9% • Detailed HPV knowledge: Mean 11.31/16 (±2.86); 41.2% high knowledge • Vaccine-specific knowledge: Notably lower (mean 5.51/11 ±1.77); 47.4% low knowledge • Primary info source: medical education (85.8%)	Interest in vaccination: 69.5%	22.8%	• Perceived unnecessary (45.3%) • Not sexually active (41.3%) • Safety concerns (34.7%) • Insufficient information (28.0%) • Lack of doctor recommendation (20.0%)	• Academic year: Higher years (4th-5th) significantly predicted uptake (OR = 4.28-5.92, p < 0.05) • Vaccine knowledge: Each unit increase in knowledge score increased odds of vaccination by 1.36-fold (p < 0.001). • Age was not a significant predictor.

The reviewed literature encompassed a diverse range of population groups: parents (n = 10) [14,15,17,19,20,21,24,29,30], university students (n = 6) [3,4,10,11,23,30], women from the general public (n = 6) [5,6,12,13,16,28], healthcare professionals (n = 4) [7,8,22,24], and female patients (n = 4) [9,11,18,27]. All studies utilized a cross-sectional design, with one incorporating a pre-post educational intervention [4]. Data collection was predominantly performed via self-administered questionnaires, with a notable increase in the use of online surveys in more recent publications [12,13,21,24,25,28].

Methodologically, 16 studies assessed both knowledge and acceptance attitudes [5,6,9,10,12,13,15,16,18,19,21,25,27,28,29,30], 8 evaluated knowledge, attitudes, and actual vaccination uptake [7,8,11,14,17,20,22,24], and six focused primarily on knowledge assessment [3,4,23,26]. Regarding vaccination targets, 22 studies examined attitudes toward female vaccination exclusively [3-6,9,11,14,16,18,20,22,24,26,27,29,30], while eight studies examined attitudes toward vaccination for both genders [7,8,10,12,13,15,17,28].

Knowledge, Acceptance, and Uptake: A Critical Gap

A synthesis of the key outcomes, organized by population group, is presented in Table 2. The analysis reveals a critical disconnect between knowledge, theoretical acceptance, and actual vaccination behavior. Knowledge levels were profoundly low among the general public and parents, with awareness of HPV ranging from 10.5% to 59.3% [5,9,26]. A stark disparity was observed between medical students, who demonstrated high awareness (>80-95%), and non-medical students, who showed significant gaps (20-40%) [10,11]. Healthcare professionals exhibited variable knowledge, with specialists like obstetrician/gynecologists showing the highest proficiency [7]. 

**Table 2:** Synthesis of key outcomes by population group

Population group	Studies	Summary of knowledge levels	Range of acceptance (%)	Range of actual uptake (%)	Most common predictors of acceptance
University students	6 [3,4,11,12,24,31]	Extreme disparity: Medical students show high awareness (>80-95%) [11,31], while non-medical students demonstrate very poor knowledge (20-40%) [11,12]. Gaps exist in knowledge of specifics (dosing, age) [12,24].	Personal: 26.7-72.0 [11,12]	Personal: 2.1-22.8 [12,31]	Enrollment in medical curriculum [11,31], higher academic year [24,31], prior knowledge of HPV/cervical cancer [12,31]
Healthcare professionals	4 [8,9,23,25]	Moderate to high, but highly variable. Obstetrician/gynecologists show the highest proficiency [8]. Non-specialists and pediatricians have significant knowledge gaps and misconceptions [8,9].	Personal: 41.2 [8]. Parental (for patients/children): 66.5-87.9 [9]. Parental (own children): 77.6-82.1 [8,25]	Personal: 3.5-7.6 [8,9]	Ob/Gyn specialty [8], younger age (<40 years) [8], adequate knowledge score [8,9], working in the private sector [8]
Women (general public)	6 [6,7,13,14,17,29]	Very low to poor. Mean knowledge scores are often below 50% [10,13]. A significant portion (up to 41%) score zero correct answers [10]. Awareness of the vaccine's existence is a primary barrier [6,13,29].	Personal: 37.5-67.6 (often cost-dependent) [17,29]. Parental (for daughters): 29.1-86.5 [10,13,14]	Personal: 1.0-3.4 [12,13,28]. Screening (Pap smear): ~2.0 [6]	Higher education and income [13,17,29], urban residence [14], married status [13,13], positive attitude toward preventive health [12], undergoing Pap smears [10,29]
Parents	8 [15,16,18,20,21,22,25,30]	Very low to poor. Widespread lack of awareness is the norm [16,17,20,27]. Major gaps exist in understanding what HPV is, how it spreads, and the purpose of the vaccine [21,30].	For children: 29.1-86.5 (many studies cluster in the 40-65% range) [10,15,18,20,22,30]	Child uptake: 7.2-10.5 [15,16,18]	Higher parental education (especially mother's) [18,20,22,30], female gender of respondent [20,22,30], receiving a recommendation from a healthcare provider [18,25,30]

Despite these knowledge deficits, theoretical acceptance rates were often moderate to high. Healthcare professionals reported a 77.6-87.9% willingness to recommend the vaccine [8], and parental acceptance for daughters ranged from 29.1% to 86.5% [9,12,13]. In stark contrast, actual vaccination uptake was critically low across all groups, ranging from 1% to 23% [7,8,11,12,13,28,30], highlighting a severe knowledge-to-action gap.

Barriers to HPV Vaccination

The primary barriers to vaccination, categorized and ranked by frequency across the studies, are detailed in Table 3. The most significant obstacle was a profound Knowledge & Awareness Deficit, cited by over 15 studies. This was followed by Safety Concerns & Fear (approximately 12 studies) and Low Perceived Risk & Need (approximately 10 studies). Systemic & Access Issues (e.g., lack of provider recommendations, cost) and Cultural & Attitudinal barriers were also identified as significant impediments. 

**Table 3:** Categorization and frequency of primary barriers to HPV vaccination

Barrier category	Specific barriers	Frequency (No. of Studies Citing)
Knowledge & Awareness Deficit [3], [6], [7], [8], [10], [14], [15], [18], [19], [25], [27], [29], [30]	"Lack of knowledge/information", "Never heard of vaccine", "Unaware of personal risk", "Didn't know where to get information/vaccine", "Unaware vaccine is offered for free by the MOH"	Very High (>15)
Safety Concerns & Fear [3], [7], [8], [12], [17], [19], [21], [25], [28], [29], [31]	"Worry/fear of side effects", "Vaccine is too new", "Uncertain about long-term effects", "Concern about vaccine safety", "Fear of injection/needles"	High (~12)
Low Perceived Risk & Need [3], [8], [16], [17], [19], [22], [27], [28], [31]	"Not at risk", "Not sexually active", "Cancer is rare/not a threat", "Belief that antibiotics can treat HPV", "Perception of being too old for vaccination"	High (~10)
Systemic & Access Issues [3], [6], [7], [10], [11], [18], [25], [29]	"Lack of doctor/healthcare provider recommendation", "Cost" (when unaware of free program), "Not available in local primary healthcare center", "Time constraints"	Moderate (~8)
Cultural & Attitudinal [3], [7], [9], [19], [22], [27]	"Fear of injection", "Family/spousal refusal", "Belief it encourages promiscuity/early sexual activity", "Embarrassment/shyness", "Preference for natural immunity"	Moderate (~7)

Predictors of Vaccine Acceptance

Table 4 consolidates the significant predictors of HPV vaccine acceptance and uptake. The analysis identified higher education level (very strong evidence from >10 studies) and employment/affiliation in the healthcare sector (very strong evidence from >8 studies) as the most powerful predictors. Adequate HPV-related knowledge was itself a strong predictor of acceptance. Other significant factors included female gender (of the respondent), younger age, higher income, and critically, recommendation from a healthcare provider.

**Table 4:** Consolidated significant predictors of HPV vaccine acceptance/uptake

Predictor	Predicts:	Strength of Evidence (No. of Studies)
Higher education level [6], [14], [17], [18], [23], [26], [28], [29]	↑ Knowledge, ↑ Acceptance, ↑ Uptake	Very Strong (>10)
Employment/affiliation in healthcare [3], [8], [11], [14], [30], [31]	↑ Knowledge, ↑ Acceptance, ↑ Uptake	Very Strong (>8)
Adequate HPV-related knowledge [8], [14], [30], [31]	↑ Acceptance, ↑ Uptake	Strong (~7)
Female gender [10], [20], [21], [23], [26], [27]	↑ Knowledge, ↑ Acceptance (for children)	Strong (~6)
Younger age (< 40-50 years) [8], [12], [18], [24], [26]	↑ Knowledge, ↑ Positive Attitude	Moderate (~5)
Recommendation from a healthcare provider [6], [18], [25], [30]	↑ Acceptance, ↑ Uptake	Moderate (~4)
Higher income [17], [28], [29]	↑ Knowledge, ↑ Acceptance	Moderate (~4)

Discussion

This systematic review of 28 studies highlights a critical prevention paradox in Saudi Arabia's public health: high theoretical acceptance of HPV vaccination contrasts with alarmingly low actual uptake. The "know-do gap" between willingness (77.6-87.9% among healthcare professionals for their children) and action (1-23% uptake across populations) shows that intention alone is insufficient [8,9,12,13,14,29,31]. A fundamental knowledge deficit is identified as the most frequent barrier to vaccination [3,6,7,8,10,14,15,18,19,25,27,29,30]. Widespread unawareness of HPV, its link to cancer, and the vaccine’s availability presents a foundational barrier that must be addressed.

Our findings reveal significant health inequities, with higher education and employment in healthcare being strong predictors of knowledge and acceptance [3,6,8,11,14,17,18,23,26,28,29,30,31]. This highlights the social stratification of access to health information, disproportionately disadvantaging vulnerable populations. Adequate HPV-related knowledge is a strong predictor of vaccine acceptance [9,15,30,31], stressing the need for targeted education to boost uptake.

A critical failure identified is the lack of engagement by healthcare providers. Providers often do not advertise for the vaccine, a significant missed opportunity, as their endorsement strongly influences acceptance [18,25,30]. Knowledge gaps among non-specialist professionals [8,9] indicate a pressing need for both public education and provider training. Additionally, barriers such as safety concerns and low perceived risk [3,7,8,12,17,19,21,25,28,29,31] stem from an underlying information deficit and specific cultural factors [3,7,9,19,22,27].

The evidence suggests that low HPV vaccination rates reflect a systemic failure rather than simply public hesitancy. Even with the vaccine being free, the gap between willingness and actual uptake appears to be influenced by fears of social stigma, such as concerns that vaccination implies early sexual activity or contradicts cultural norms. To overcome this, we recommend implementing mandatory HPV vaccination for schoolgirls, a strategy proven effective in the past with vaccines like DPT. Historically, making routine vaccines compulsory successfully translated high public willingness into tangible uptake. A coordinated national strategy should also include awareness campaigns to address misinformation and social concerns, as well as mandatory education for healthcare providers to ensure consistent, supportive messaging.

Comparison With Regional and Global Context

Our findings align with a consistent regional pattern, corroborated by Alshahrani et al.'s GCC review, which highlights a significant lack of parental knowledge and safety concerns as critical barriers across Gulf nations [1]. This consistency underscores the validity of our results and indicates shared cultural and health system issues.

In addition, the Eastern Mediterranean Region (EMR) meta-analysis by Gebreal et al. (2025) [2] contextualizes our findings. Their reported parental willingness (61%) and actual uptake (7%) closely match our Saudi-specific review (29.1-86.5% willingness; 7.2-10.5% uptake) [10,13,14,15,16,18,20,22,30]. This stark discrepancy highlights a significant public health crisis. The Gebreal et al. study emphasizes that knowledge drives willingness (rising to 75% with adequate understanding) and that healthcare provider recommendations are crucial predictors [2].

The key difference between these analyses lies in depth and specificity. While the GCC and EMR reviews confirm widespread challenges, our comprehensive Saudi-specific study reveals a nuanced understanding of the issue. We explore the "know-do gap" across various demographics, identify a broader range of predictors (e.g., education, gender, income), and categorize barriers to inform targeted national interventions. Whereas the EMR study addresses general factors that impact variance, ours provides essential insights for effective national policy-making. Collectively, these reviews illustrate a regional challenge that demands coordinated responses tailored to specific national needs.

The EMR meta-analysis affirms that our Saudi-specific findings are a critical piece of a larger regional puzzle. The consistency across reviews underscores the urgent need for the coordinated strategy we propose. The regional data demonstrates that success is achievable, as evidenced by high willingness rates (>80%) in subgroups with higher education, healthcare employment, and good knowledge. The challenge now is to translate this evidence into action through massive education campaigns and systemic healthcare reform to convert the vast existing pool of willingness into tangible vaccination coverage and prevent future HPV-related cancers.

Clinical and Public Health Implications

This review highlights that Saudi Arabia has largely overcome the initial "know-how" barrier regarding HPV vaccination, with the vaccine now freely accessible at all government health centers and fully covered by insurance in the private sector. The challenge has shifted from awareness to implementation-the critical "know-to-do" gap. Despite available knowledge and access, uptake remains low due to persistent socio-cultural concerns, including fear of stigma and misperceptions linking vaccination to promiscuity. To address this, structured and repeated educational efforts led by healthcare providers are essential, focusing on reinforcing the vaccine's safety and its role in cancer prevention. Leveraging the credibility of health professionals and official Ministry endorsements can normalize vaccination, reduce hesitation, and translate high willingness into actual uptake, ultimately reducing HPV-related cancer rates.

Strengths and Limitations

The review’s strength lies in its rigorous adherence to PRISMA guidelines [4], with independent processes for study selection, data extraction, and quality assessment, thereby enhancing the reliability of the synthesis. The inclusion of 28 studies offers a comprehensive analysis of the Saudi context, quantifying the disconnect between acceptance and actual vaccine uptake. However, certain limitations exist. The substantial heterogeneity in study populations, measurement tools, and outcome reporting precluded meta-analysis and may impact generalizability. The predominance of cross-sectional designs establishes associations but not causality. Additionally, reliance on self-reported data raises concerns about social desirability bias, in which participants may exaggerate their willingness to vaccinate, making the low vaccination rates even more alarming.

Recommendations for Future Research

Future research should utilize longitudinal designs to track how knowledge and attitudes affect vaccine uptake over time. It is essential to include underrepresented groups, such as fathers, rural populations, and individuals with lower levels of education, to ensure equitable intervention development. The creation of standardized, validated Arabic assessment tools is urgently needed for direct comparisons and meta-analyses. Additionally, implementation science research is necessary to design, test, and measure the effectiveness of culturally tailored educational interventions in clinical and community settings, focusing on improving vaccination rates rather than just knowledge. Lastly, in-depth qualitative studies are vital to understanding the socio-cultural and religious factors influencing vaccine decision-making.

## Conclusions

This review underscores a critical prevention paradox in Saudi Arabia: while willingness to receive the HPV vaccine is encouragingly high (around 70%), actual uptake remains critically low (approximately 10%). This indicates that the primary public health priority is no longer raising awareness or willingness, but rather converting existing intention into tangible vaccination. The urgent need is to increase the 10% uptake rate, not the 70% willingness rate, by addressing persistent implementation barriers, especially sociocultural hesitations and operational gaps in delivery. A targeted national strategy should focus on actionable, system-level interventions such as mandatory school-based vaccination programs, strengthened provider recommendation protocols, and public campaigns that directly counter stigma and misinformation. By shifting focus from building acceptance to enabling action, Saudi Arabia can effectively close the intention-action gap and significantly reduce the future burden of HPV-related cancers.

## References

[1] Alshahrani NZ, Alshahrani JA, Almushari BS (2024). Parental perspectives on human papillomavirus (HPV) vaccination in Gulf Cooperation Council Countries: a systematic review. Medicine (Baltimore).

[2] Gebreal A, Ashmawy R, Ahmed MJ (2025). A systematic review and meta-analysis on parental uptake and willingness to vaccinate children against human papillomavirus in the Eastern Mediterranean Region. Vaccine.

[3] Al-Shaikh GK, Almussaed EM, Fayed AA, Khan FH, Syed SB, Al-Tamimi TN, Elmorshedy HN (2014). Knowledge of Saudi female university students regarding cervical cancer and acceptance of the human papilloma virus vaccine. Saudi Med J.

[4] Page MJ, McKenzie JE, Bossuyt PM (2021). The PRISMA 2020 statement: an updated guideline for reporting systematic reviews. BMJ.

[5] Al-Shaikh GK, Syed SB, Fayed AA, Al-Shaikh RA, Al-Mussaed EM, Khan FH, Elmorshedy HN (2017). Effectiveness of health education programme: Level of knowledge about prevention of cervical cancer among Saudi female healthcare students. J Pak Med Assoc.

[6] Malibari SS (2018). Knowledge about cervical cancer among women in Saudi Arabia. Egypt J Hosp Med.

[7] Alnafisah RA, Alsuhaibani R, Alharbi MA, Alsohaibani AA, Ismail AA (2019). Saudi women's knowledge and attitude toward cervical cancer screening, treatment, and prevention: a cross-sectional study in Qassim Region (2018-2019). Asian Pac J Cancer Prev.

[8] Anfinan NM (2019). Physician's knowledge and opinions on human papillomavirus vaccination: a cross-sectional study, Saudi Arabia. BMC Health Serv Res.

[9] Almazrou S, Saddik B, Jradi H (2020). Knowledge, attitudes, and practices of Saudi physicians regarding cervical cancer and the human papilloma virus vaccine. J Infect Public Health.

[10] Alrajeh MF, Alshammari SA (2020). Awareness of human papillomavirus and its vaccine among patients attending primary care clinics at King Saud University Medical City. J Nat Sci Med.

[11] Azer SA, AlSaleem A, Albassam N, Khateeb R, Alessa A, Aljaloud A, Alkathiri S (2022). What do university students know about cervical cancer and HPV vaccine?. Eur Rev Med Pharmacol Sci.

[12] Gari A, Alsulaimani N, Brashi R, Alharbi R, Alharbi A, Bakhsh H (2022). Awareness, knowledge, and attitude toward cervical cancer and hpv prevention among female medical school students. Cureus.

[13] Ibrahim HA, Nahari MH, Al-Thubaity DD, Alshahrani MA, Elgzar WT, El Sayed HA, Sayed SH (2022). Saudi women health beliefs and associated factors regarding cervical cancer prevention at Najran city: a theory-based study. Afr J Reprod Health.

[14] Barhamain AS, Alwafi OM (2022). Uptake of human papilloma virus vaccine and intention to vaccinate among women in Saudi Arabia. Med Sci.

[15] Alhusayn KO, Alkhenizan A, Abdulkarim A, Sultana H, Alsulaiman T, Alendijani Y (2022). Attitude and hesitancy of human papillomavirus vaccine among Saudi parents. J Family Med Prim Care.

[16] Alkalash SH, Alshamrani FA, Alhashmi Alamer EH, Alrabi GM, Almazariqi FA, Shaynawy HM (2022). Parents' knowledge of and attitude toward the human papillomavirus vaccine in the Western Region of Saudi Arabia. Cureus.

[17] Turki YM, Alqurashi J (2023). Knowledge, attitudes, and perceptions towards human papillomavirus (HPV) vaccination among adult women in primary health care centers in Makkah, Saudi Arabia. Cureus.

[18] Alnaeem L, Alanizi S, AlQarni G, Alwadani J, Bomouzah F, Ali Z (2023). Acceptance, knowledge, and attitude of parents toward the human papillomavirus vaccine in the eastern region of Saudi Arabia: a cross-sectional study. Cureus.

[19] Tobaiqy MA, Mehdar SA, Altayeb TI, Saad TM, Alqutub ST (2023). Parental knowledge, views, and perceptions of human papilloma virus infection and vaccination-cross-sectional descriptive study. J Family Med Prim Care.

[20] Alshehri MA, Fahim WA, Alsaigh RR (2023). The association between parents' knowledge about human papillomavirus and their intention to vaccinate their daughters: a cross-sectional study. Cureus.

[21] Maqbul MS, Allihaydan FS, Elfaham RH, Aljohani WA, Alharbi MS, Alsuwat MA, Aljohani RA (2024). Perceptions, attitude, and knowledge of Saudi parents towards the human papilloma virus vaccine. Vacunas.

[22] Bin Alamir AA, Almotairi AH, Almutairi FH (2024). Knowledge and attitude of parents regarding the human papillomavirus vaccine as a new component in the Saudi vaccination schedule. Cureus.

[23] Alherz FA, Alamri AA, Aljbreen A, Alwallan N (2024). Knowledge of cervical cancer, human papillomavirus (HPV), and acceptance of the HPV vaccine among parents of daughters in Riyadh, Saudi Arabia. J Infect Public Health.

[24] Aldawood E, Alzamil L, Dabbagh D, Hafiz TA, Alharbi S, Alfhili MA (2024). The effect of educational intervention on human papillomavirus knowledge among male and female college students in Riyadh. Medicina (Kaunas).

[25] Bakhashab AS, Aljilani SA, Alkinaidri NM (2025). Knowledge, attitude, and perception of the parents toward HPV vaccine administration to their children in Saudi Arabia: a cross-sectional study. Front Public Health.

[26] Algaadi SA, Aldhafiri HJ, Alsubhi RS, Almakrami M, Aljamaan NH, Almulhim YA (2024). The Saudi population's knowledge and attitude towards human papillomavirus (HPV) infection and its vaccination. Cureus.

[27] Alshrari AS (2024). Public knowledge, attitude, and perception of human papilloma virus in northern border province, Saudi Arabia: a cross-sectional study. Risk Manag Healthc Policy.

[28] Tobaiqi MA, Albouq RA, Ban AM (2024). Knowledge, awareness, and attitudes towards HPV and its vaccination among women in the Medina region: a cross-sectional study. Healthcare (Basel).

[29] Azzi A, Al-Dhelaan R, Al-Fahd A, Almukhalafi O, Al-Tayar N, Abutheeb A, Bahmid M (2024). Assessment of awareness level among women about cervical smears, human papillomavirus (HPV), and HPV vaccine in Saudi Arabia. PLoS One.

[30] Alshahrani MN, Almutairi D, Zahrani Y, Alsabaani A, Alraey Y, Alshahrani SM (2025). Barriers associated with the parental acceptance of human papillomavirus (HPV) vaccination of minors in Asir, Saudi Arabia: a cross-sectional study. Hum Vaccin Immunother.

[31] Bakhsh H, Ali Altamimi S, Aldosari FN (2025). Barriers and predictors of HPV vaccine uptake among female medical students in Saudi Arabia: a multi-center cross-sectional study. Healthcare (Basel).

